# Investigation on Surface Quality of a Rapidly Solidified Al–50%Si Alloy Component for Deep-Space Applications

**DOI:** 10.3390/ma13153412

**Published:** 2020-08-03

**Authors:** Oussama Chaieb, Oluwole A. Olufayo, Victor Songmene, Mohammad Jahazi

**Affiliations:** Department of Mechanical Engineering, École de Technologie Supérieure (ÉTS), Montreal, QC H3C 1K3, Canada; oussama.chaieb.1@ens.etsmtl.ca (O.C.); oluwole-ayodeji.olufayo.1@ens.etsmtl.ca (O.A.O.); Mohammad.Jahazi@etsmtl.ca (M.J.)

**Keywords:** rapidly solidified aluminum, high silicon, machining, milling, surface finish, microstructure change

## Abstract

To meet the requirements for high-performance products, the aerospace industry increasingly needs to assess the behavior of new and advanced materials during manufacturing processes and to ensure they possess adequate machinability, as well as high performance and an extensive lifecycles. Over the years, industrial research works have focused on developing new alloys with an increased thermal conductivity as well as increased strength. High silicon content aluminum (Al–Si) alloys, due to their increased thermal conductivity, low coefficient of thermal expansion, and low density, have been identified as suitable materials for space applications. Some of these applications require the use of intricate parts with tight tolerances and surface integrity. These challenges are often tied to the machining conditions and strategies, as well as to workpiece materials. In this study, experimental milling tests were performed on a rapidly solidified (RS) Al–Si alloy with a prominent silicon content (over 50%) to address challenges linked to material expansion in deep space applications. The tests were performed using a polycrystalline cubic boron nitride (PCBN) tool coated with amorphous diamond to reduce tool wear, material adhesion, surface oxidation, and particle diffusion. The effects of cutting parameters on part surface roughness and microstructure were analyzed. A comparative analysis of the surface with a conventionally utilized Al6061-T6 alloy showed an improvement in surface roughness measurements when using the RS Al–Si alloy. The results indicated that lower cutting speed and feed rate on both conventional and RS Al–Si alloys produced a better surface finish. Reduced vibrations were also identified in the RS Al–Si alloy, which possessed a stable cutting time at low cutting speeds but only displayed notable vibrations at cutting speeds above 120 m/min.

## 1. Introduction

**Figure 1:**
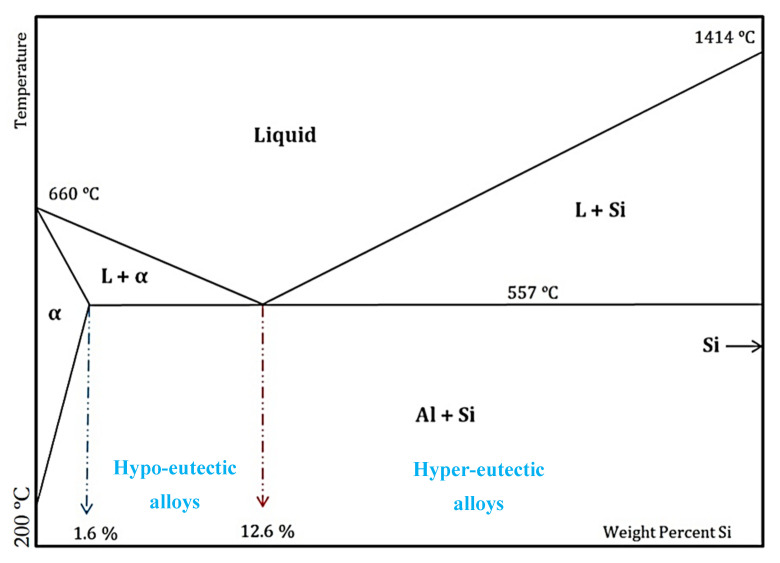
The schematic phase diagram of Al–Si [4].

**Figure 2:**
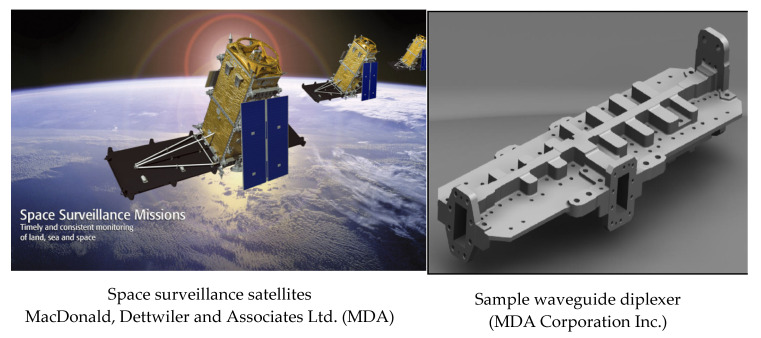
Some applications of rapidly solidified aluminum alloys [5].

The performance needs of products are ever-evolving. The industry is increasingly faced with the need to reassess its manufacturing process flow to match demand. Industrial changes in manufacturing processes encompass material property changes and production methodologies, as well as the interrelation of these factors to ensure high performance in operation. Over the years, automotive and aerospace research works have focused on developing new alloys with increased thermal conductivity, increased strength, and lower thermal expansion [1,2]. High silicon content aluminum (Al–Si) alloys possess this combination of unique properties, including a high strength, a high wear resistance, a low thermal expansion, good castability, and good vibration dampening [3]. These aluminum alloys, which possess a silicon content higher than 12.6%, are termed hypereutectic. This name is derived due to their occurrence above the eutectic point at a composition of about 12% and a temperature of 633 °C (Figure 1). These alloys are often used in applications where high strength and low thermal expansion are required, such as in automotive components (cylinder heads, clutch housing, compressors, and engine blocks) and deep space applications (waveguide diplexers and microwave components) (Figure 2).

### Machinability of High Si–Al Alloys

Aluminum alloys have a long and fruitful history in aerospace. These materials have seen applications in frames, fuselage, wings, and engine parts. Various forms of aluminum alloys with different mechanical properties, emanating from the combination with different elements such as zinc, copper, silicon, and magnesium, have been developed. As seen from the literature, high silicon content Al–Si alloys possess good strength characteristics, higher fatigue limits, and excellent wear resistance suitable for aerospace component [3]. Despite the various advantages of Al–Si alloys for deep space aerospace applications, these alloys still face challenges in machining during production. These problems are related to their chip formation, microstructural integrity, and the generation of a desired surface finish. These challenges are often tied to the machining conditions and strategies employed but they also could be because of the selected workpiece materials and cutting tools used. The elevated addition Si-content into these aluminum alloys increases the difficulties faced in machining due to the increase in the abrasive load between the tool and the workpiece. This is explained by the hard nature of the silicon particles within the aluminum matrix, thus accelerating the abrasive wear on the tool in combination with its adhesive effect and chemical reactions [3]. Furthermore, such high Si-content alloys limit the usable cutting speed in the higher range due to the emergence of defective chip formation, which in turns decrease surface quality [6] because such materials react like metal matrix composites. The chips formed during the machining of these materials also break more easily with the increase of silicon [3], similar as the action of reinforcements in aluminum metal matrix composites, as denoted by Songmene and Balazinski [7]. This chip breaking phenomena is also exacerbated at some lower cutting speed values [8].

In a study on the tool life when machining high silicon content Al–Si, König and Erinski [6] reported that the tool life of hypereutectic alloys with 17% silicon is lower than all tool lives when machining other aluminum–silicon casting materials. Their study also indicated the adverse effect of an increase in Si-content towards high surface integrity. However, a study conducted by Akyüz [9] on the effect of Si-content on the machinability of Al–Si alloys revealed contradicting results. In his study on four alloys with different ratios of Si content ranging from 2% to 12%, Akyüz [9] observed an improved surface quality with an increase in Si percentage content. This could however reflect the importance of the percentage silicon present in the alloys. Another study by Ma et al. [10] indicated an increase in mechanical strength and a decrease in elongation with Si content from 12 to 35 wt%, [10]. Their results were characterized by shorter chips due to increased brittle nature of the alloy. They also observed a fall in the coefficient of thermal expansion (CTE) with rising Si content due to the restriction of Si on the aluminum matrix. These studies showed possible advantages that could also be obtained with a higher percentage content of silicon in these aluminum alloys. Besides the influence of Si-content on surface condition, a study into its influence on cutting parameters selection by Lin et al. [11] on the machining of a 20% silicon of carbide-reinforced aluminum metal matrix composite identified the feed rate as the most significant parameter to surface roughness. The results revealed an improvement in surface quality and tool life with lower feed rates. 

Furthermore, the manufacturing process of high Si-content aluminum alloys has been shown to influence the mechanical properties and microstructure of the alloy. Different methodologies have been employed in creating these high silicon content aluminum alloys. Tanaka and Akasawa [12] studied the machining differences between conventionally processed ingot metallurgy aluminum alloy and powder metallurgy using a rapid solidification process. In their results, improved flank wear, surface roughness, and cutting force were found when machining the rapidly solidified alloy; however, they indicated a need to control chip breaking. The study into high silicon content aluminum alloys (Al–Si) to meet the requirements for high-performance component for deep-space applications is essential for long-lasting satellites. However, additional research into the manufacturing process and machining conditions used in the production of these materials for deep space components is needed.

## 2. Importance of Surface Quality in the Production of Satellite Components

Most receiving antennas introduce phase errors of their own because of small mechanical errors in their structure, which defines the phase front of the antenna. These may arise from machining tolerances, errors in antenna structural adjustment, and surface roughness from production [13]. Surface roughness is an important parameter for these antennas, especially for components operating in high-frequency ranges such as mmW and THz, because a rough surface could degrade the electromagnetic performance in terms of increased conductor loss [14]. For these radio frequency networks of precision microwave components ranging from 1 to 90 GHz, precise physical overall dimensions, close mechanical tolerances, and single-piece construction result in increased performances. Internal polished waveguide surfaces result in lower insertion losses. Manufacturing techniques used in the production of satellite components thus demand improved high-quality standard materials and optimized cutting processes to mitigate the adverse effect errors hold on product functionality.

This study sought to evaluate the possible use of a new low CTE aluminum alloy with improved mechanical properties such as high strength and wear resistance found in the 6061-T6 and 7075-T6 alloys but with a lower CTE. However, because this innovative alloy contains a higher proportion of silicon than most used hypereutectic alloys (around 50%), a research gap exists in identifying the adequate machining parameters to generate a high surface profile for production. Furthermore, the need to establish the machining behavior and practices for this material conventional milling is needed.

## 3. Experimental Setup

### 3.1. Material Characterization

**Figure 3:**
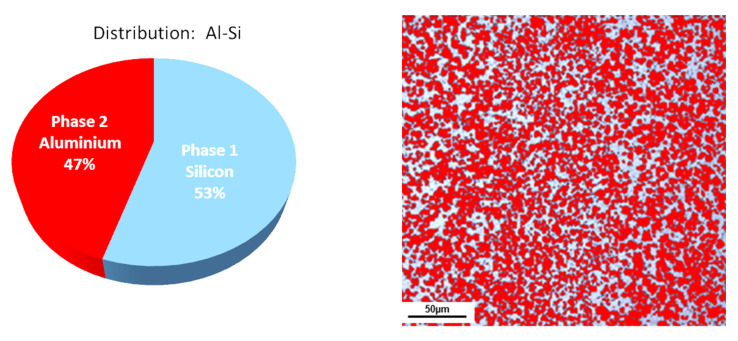
Volume fraction of elements in the hypereutectic rapidly solidified (RS) alloy.

For the experimental study, a rapidly solidified hypereutectic aluminum alloy produced by melt-spinning, extrusion, and ultra-fast cooling was manufactured by Advanced Materials Technology (AMT) in Germany was used. A volume fraction analysis of the alloy was performed, and the following phase proportions were obtained: 53% silicon and 47% aluminum (as shown in Figure 3). 

The advantages of the rapid solidification process on material property enhancements have been seen in numerous research studies [15]. At cooling rates in the order of 10^4^–10^7^ K/s, a fine grain microstructure with an increased strength is produced. The cooling rate has a noticeable effect on the size, morphology, and distribution of all the microstructural constituents. An increase in the cooling rate refines all microstructural features in size, decreases secondary dendrite arm spacing, changes the morphology of the eutectic Si from large and elongated plates-like to small and rounder ones, and decreases the size of all intermetallic compounds [4]. The use of rapidly solidified Al–Si alloys in industrial applications under adequate machining conditions can provide industries with a suitable light-weight alloy with improved mechanical properties [16].

**Figure 4:**
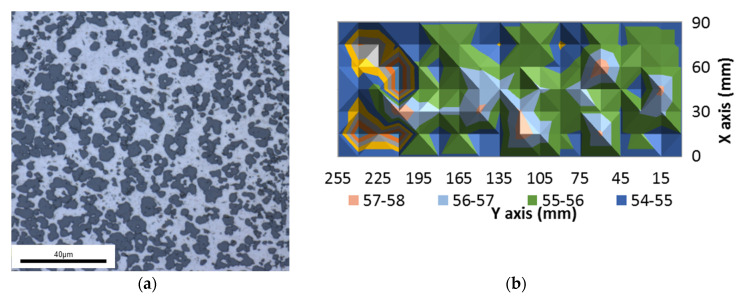
(**a**) Microstructure of hypereutectic RS Al–50%Si alloy and (**b**) HRB hardness distribution.

The material microstructure consists of an Al matrix characterized by eutectic silicon particles with some denser zones. An evaluation of the hardness distribution of the material was conducted to provide a viewpoint on the homogeneity of the material. An average hardness of 54 ± 4 Rockwell Hardness (HRB) was determined for the sample for both traverse and longitudinal cuts (Figure 4b). The Brinell superficial hardness was measured using a Buehler hardness tester with a 1/16 in. (1.59 mm) diameter ball and a 15 kg load. 

### 3.2. Tensile Tests

**Figure 5:**
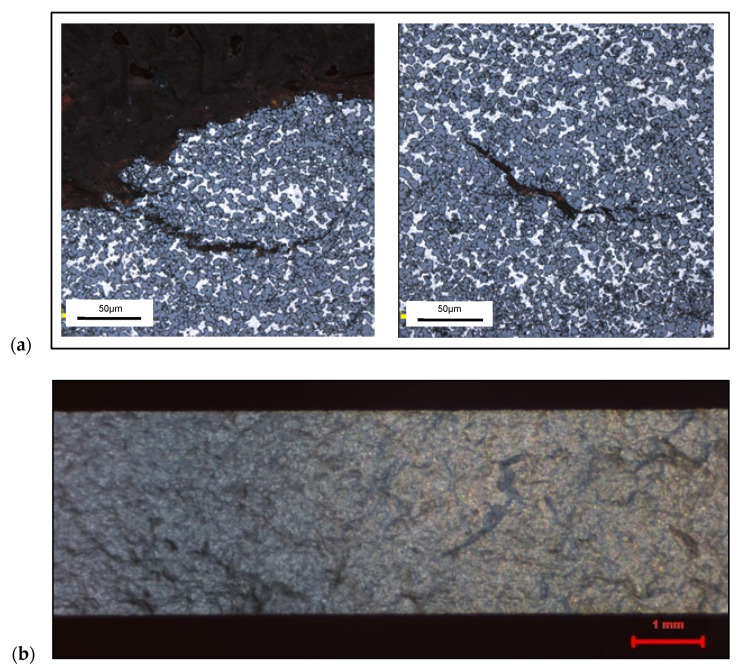
(**a**) Cracking under the effect of the tensile force on the RS Al–50%Si alloy, and (**b**) the cracked surface on the alloy sample.

Tensile tests were performed following the American Society for Testing and Materials (ASTM) E8 standards [17]. These tests were used to determine the alloy’s ultimate tensile strength, fracture strength, maximum elongation, and reduction in area (Figure 5 and Table 1).

The dense pockets of silicon observed by analyzing the microstructure were seen to harm the tensile strength. Figure 5a,b shows cracks that propagated in dense areas and caused the tearing of the material. This indicated that a large number of silicon grains increased the number of grain boundaries and thus facilitated the propagation of cracks at the bottom of the material. Table 1 shows the determined mechanical properties of the tested RS Al–50%Si material. An overview of the comparison of its mechanical properties with other hypereutectic RS Al–50%Si alloys is shown in Table 2.

### 3.3. Properties of the Cutting Tools 

**Figure 6:**
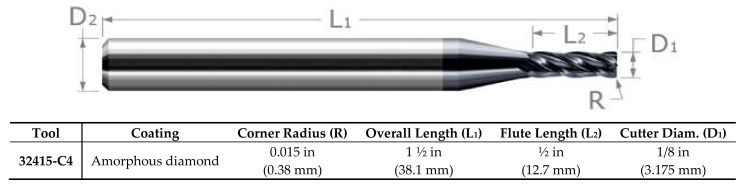
Polycrystalline cubic boron nitride (PCBN) tool coated by PVD amorphous diamond.

The tests were performed using polycrystalline cubic boron nitride (PCBN) tools coated with Physical vapor deposition (PVD) amorphous diamond to reduce the effect of wear, material adhesion, surface oxidation, and particle diffusion. The characteristics of the tools are shown in Figure 6. Five different cutting tools with a variation of 5% in tool diameters (D_1_) were selected in the course of the study to assess the effect of tool diameter on surface roughness output.

### 3.4. Milling Experiments

**Figure 7:**
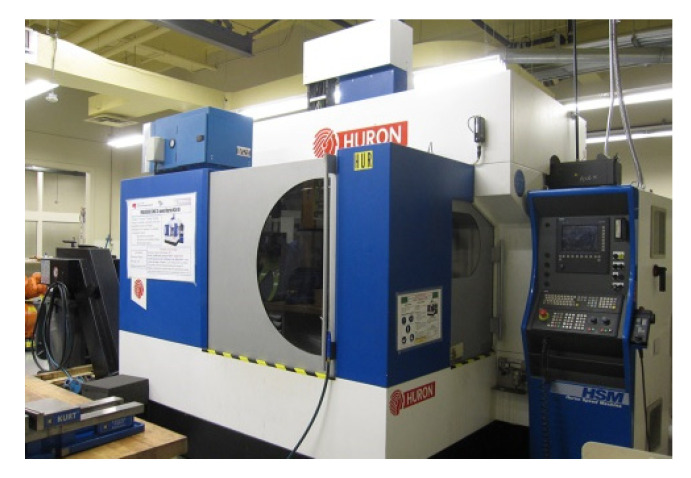
HURON K2X10 CNC milling machine.

Experimental milling tests were performed using the HURON K2X10 CNC machine (with 28,000 RPM) (Figure 7). The effects of cutting parameters such as cutting speed and feed rate on the surface roughness and vibration of the machine were also analyzed. An ICP^®^ Triaxial Accelerometer mounted on the workpiece was used according to the back-to-back comparison (AT401-3) method, with a sensitivity of 1.037 mV/m/s^2^ along the x-axis, 1.022 mV/m/s^2^ on the y-axis, and 1.022 mV/m/s^2^ along the z-axis for the 100.00 Hz frequency. 

A full factorial experiment plan including cutting speed, feed rate, depth of cut (both radial and axial), and cutting diameter as variables was implemented. The initial parameters for the cutting tool diameter were selected based on the tool supplier’s recommendation for hypereutectic materials (with Si > 12%). The designed plan consisted of a five-level step with a 5% deviation from the initial recommended cutting speed, feed rate, and tool diameter (Table 3). Modifications in the depth of cut values were also recommended by the tool manufacturer. With the five levels for the feed rate and cutting speed, 25 experimental tests were performed for each cutting tool.

### 3.5. Analysis of Surface Roughness

**Figure 8:**
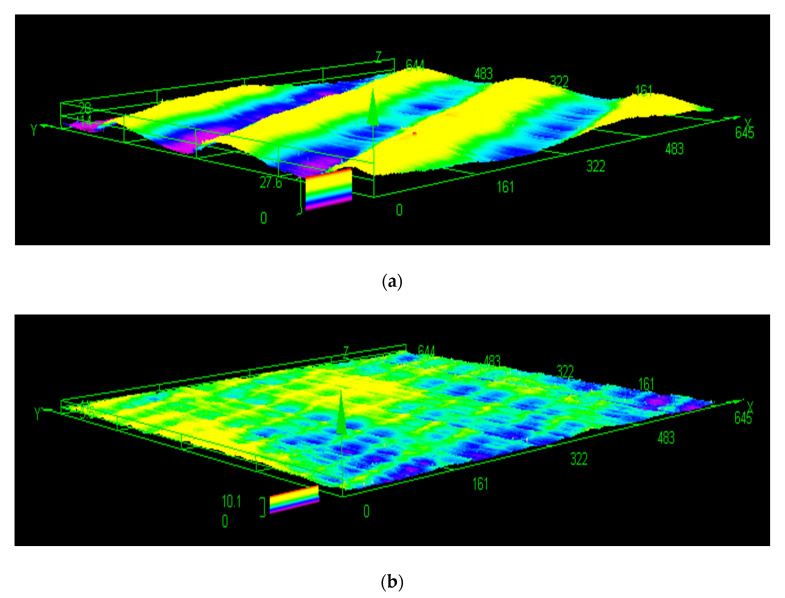
Surface integrity of a (**a**) 137.16 mm/min and 0.0254 mm/tooth experiment and a (**b**) 76.2 mm/min and 0.02032 mm/tooth experiment.

To determine the quality of the surface, a comprehensive analysis of the surface roughness parameters was performed to gain a more in-depth understanding of both average and physical roughness characteristics. The roughness of the machined surface was measured using a laser confocal microscope (OLYMPUS OLS4100, LEXT). The microscope captures various data points over a millimeter square surface area to estimate the average roughness value at the desired location. The parameters and settings used for the microscopy were obtained from previous studies [18]. Figure 8 shows two examples of surface roughness profiles of two RS Al–50%Si samples machined with extreme cutting conditions.

Figure 8a shows ripples on the machined surface at higher cutting speed of 137.16 mm/min and a feed rate of 0.0254 mm/th. However, Figure 8b shows ripple-free roughness peaks with a cutting speed lower than 76.2 mm/min and a tooth feed equal to 0.02032 mm/th. Therefore, by analyzing the data between these two cutting speed values and feed rate, we could determine the optimal cutting parameters for the best surface condition.

As defined in the study by Blateyron [19], most profile parameters have a mathematical expression that can easily convert 2D profiles into 3D. Therefore the line parameters “$R_{x}$” of roughness acquired were extended into 3D profiles “$S_{x}$” using Equation (1) below [20]:

(1) $$R_{a}=\sqrt{\frac{1}{lb}\int_{lb}Z^{2}\left(x\right)dx};\;\;S_{a}=\sqrt{\frac{1}{A}\int \int_{A}Z^{2}\left(x,y\right)dxdy\;}$$

where Z is the amplitude, *A* is the surface area, *x* and *y* are the axes, and *lb* represents the line points.

Therefore, *Sa* represents the extension of *Ra* to a surface. It shows as a single value, the difference in height of each point compared to the arithmetical mean of the surface. Additional roughness parameters *Sq, Sv, Sz, Ssk, Sku*, and *Sp* were also directly obtained in the same way with a simple extrapolation to the plane. *Sq* is defined as the root mean square value of other values within the definition area. It is also represented as the standard deviation of heights. *Sv* is the absolute value of the height of the lowest valley in an area, while *Sp* is the height of the highest peak within the defined area and *Sz* is the sum of these two distances, i.e., the distance from the largest peak to the lowest valley in that area. *Ssk* values represent the degree of bias or tilt tendency of the roughness shape, and *Sku* value relates to its sharpness of the peak profile. 

The parameter equations use integrals instead of sums because they represent the definition of a continuous case. The section below discusses the roughness and vibration results obtained from experimentation.

## 4. Results and Discussion

**Figure 9:**
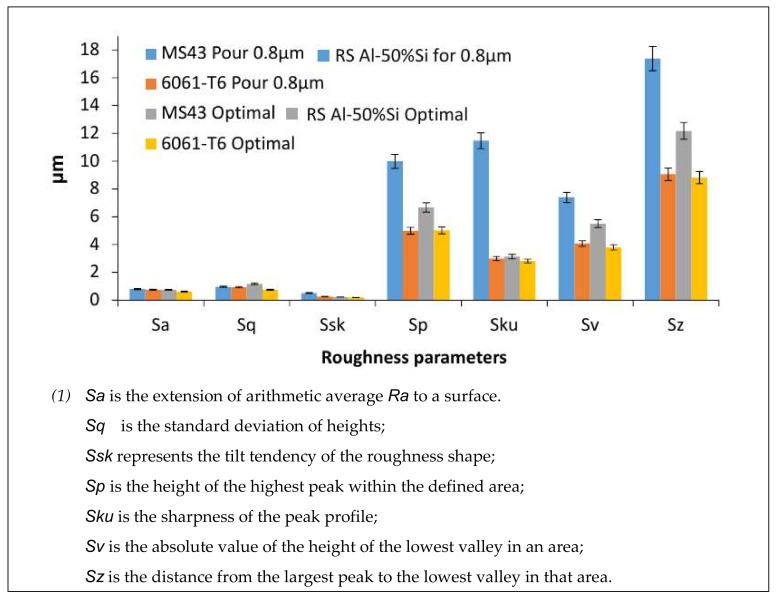
Comparison of different parameters (^1^) of surface roughness for the two alloys (Tool 3).

To attain optimal surface roughness for the deep space machined parts, a maximum average roughness value of 0.8 µm was set as the threshold for acceptable parts. At this threshold value, an evaluation of the surface roughness parameters for the RS Al–50%Si alloy and conventional Al6061-T6 alloy was performed. Figure 9 presents the results of the surface that were achievable using the threshold value (i.e., 0.8 µm) for the RS Al–50%Si alloy as well as results of the conventionally utilized Al6061-T6 alloys. Each of the described roughness parameters represents one or more of the characteristics of the machined surface. Additional values of the best overall average roughness obtained during experimentation were also added to this chart for comparison. Tool 3 (D = 3.175 mm) has been used as a reference point for result analysis in this study due to its improved conditions in terms of material removal rates and surface integrity.

Figure 9 shows the variation of the different amplitude of roughness parameters for the cutting parameters. From Figure 9, it can be seen that the arithmetic roughness was very similar for both materials, although the roughness of the hypereutectic RS Al–50%Si alloy was slightly higher than the Al6061-T6 alloy. This could have been due to the presence of the hard silicon grains, as seen in the literature [21]. In addition, a 30% increase in the physical “*Sp*” and “*Sz*” roughness parameters were observed on the RS Al–50%Si sample, which could also have been due to the effect of the solid silicon grains. Table 4 shows the optimal recommended parameters found to achieve a minimum surface roughness of 0.8 µm.

**Figure 10:**
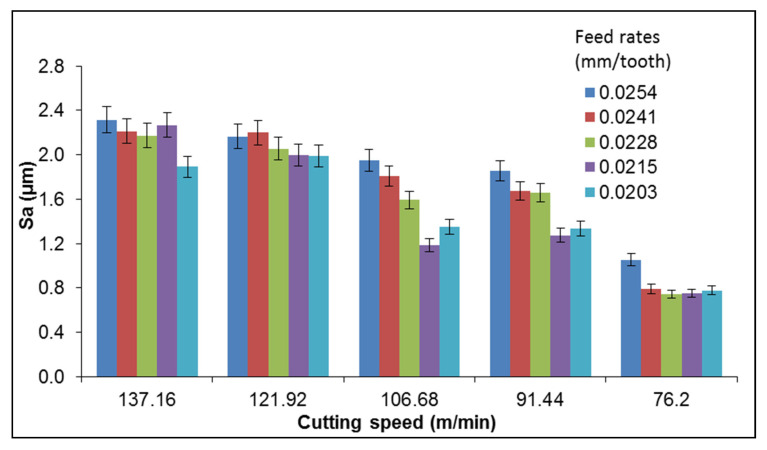
Surface roughness (*Sa*) vs. cutting speed and feed rate for the RS Al–50%Si alloy (Tool 3).

Alternatively, it can be observed that the roughness parameters were influenced by the cutting parameters. These surface roughness values acquired represent an average of several measurements made using the microscope. Figure 10 shows that the average surface roughness of the hypereutectic RS alloy was sensitive to variations in feed rate and cutting speed. In Figure 10, a rough surface is identified with increased cutting speeds of 137.16 and 121.92 m/min. Observations showed a higher impact from cutting speeds than feed rates in roughness values, with trends showing that an increase in either cutting speed or feed rate led to a corresponding increase in roughness.

### 4.1. Statistical Analysis of the Surface Roughness

A statistical evaluation of the roughness parameters “*Sa*” and “*Sq*” was performed to create a prediction model for the surface profile. The consideration of the influence the tool choice hold on the surface roughness was also analyzed.

#### 4.1.1. Analysis of Arithmetic Surface Roughness (*Sa*)

Table 5 shows the ANOVA for the transformed response for *Sa* for Tool 3. The ANOVA results were used to assess the significance of the parameters influencing the roughness response results at a 95% confidence level. The table shows that both the cutting speed and feed rate were significant factors for the roughness parameter (*Sa*). There was a 94.81% R-squared prediction accuracy of the model to the data. To obtain a power form equation of the influence of parameters, an inverse transformation of the response was performed. Table 5 shows the coefficient of the transformed response used to identify the constants of the model. The power form equation was thus obtained from the exponential of the generated regression model, as shown in Equation (2).

Model Summary for Transformed Response
S = 0.160933R-sq = 95.46%R-sq(adj) = 95.07%R-sq(pred) = 94.81%

Coefficients for Transformed Response (Tool 3)
**Term****Coef.****SE Coef*****T*-Value*****p*-Value****VIF**Speed0.5220.1264.120331.98Feed0.8220.1555.30331.98

Regression Equation:

(2) $$Sa=V^{0.522}\times f^{0.822}$$

#### 4.1.2. Analysis of Root Mean Square Roughness Parameter (*Sq*)

Likewise, the analysis of the root mean square roughness value (*Sq*) indicated that both the cutting speed and feed rate were significant terms for the model equation (Table 6). A 90.15% R-squared prediction fitting was obtained from the generated regression model to the data. The power form equation generated from the analysis is shown in Equation (3).

Model Summary for Transformed Response
S = 0.158325R-sq = 91.37%R-sq(adj) = 90.62%R-sq(pred) = 90.15%

Regression Equation:

(3) $$Sq=V^{0.527}\times f^{0.77}$$

#### 4.1.3. Regression Equations of Roughness (*Sa* and *Sq*) for the Cutting Tools 

**Figure 11:**
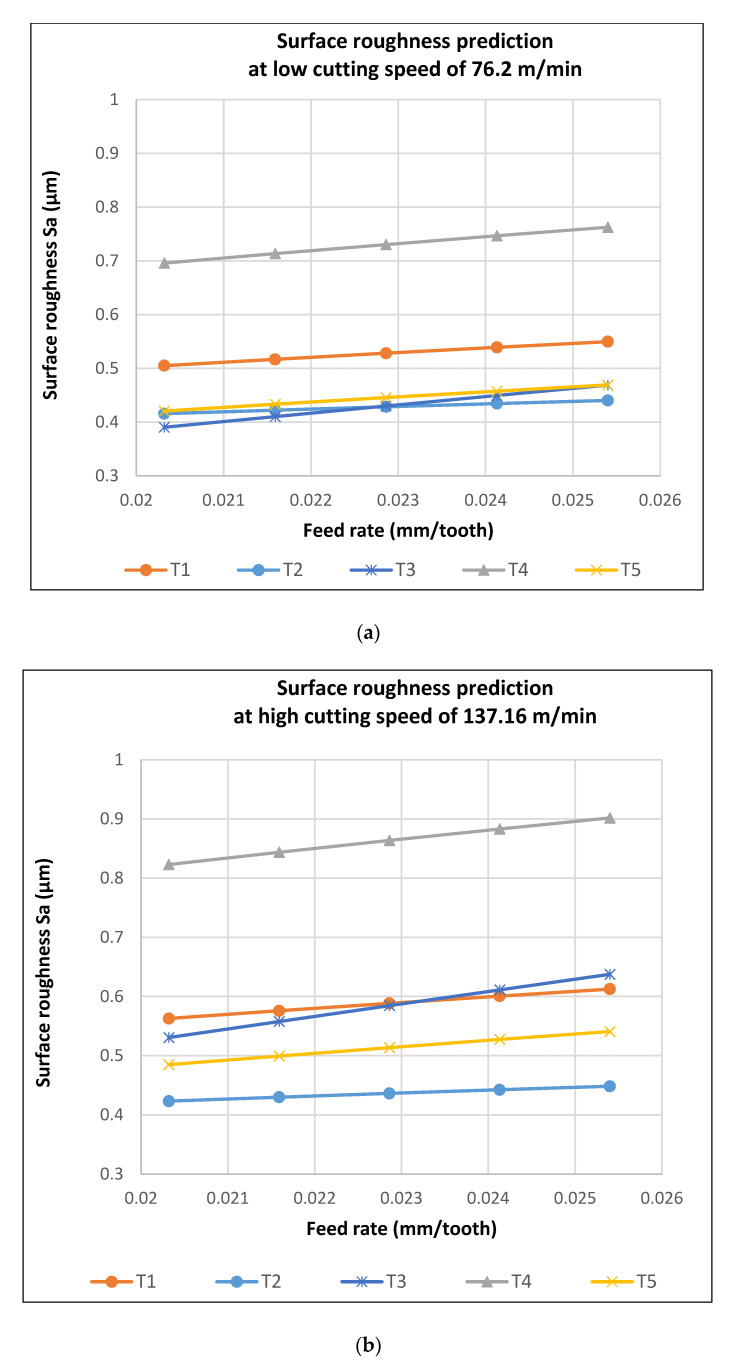

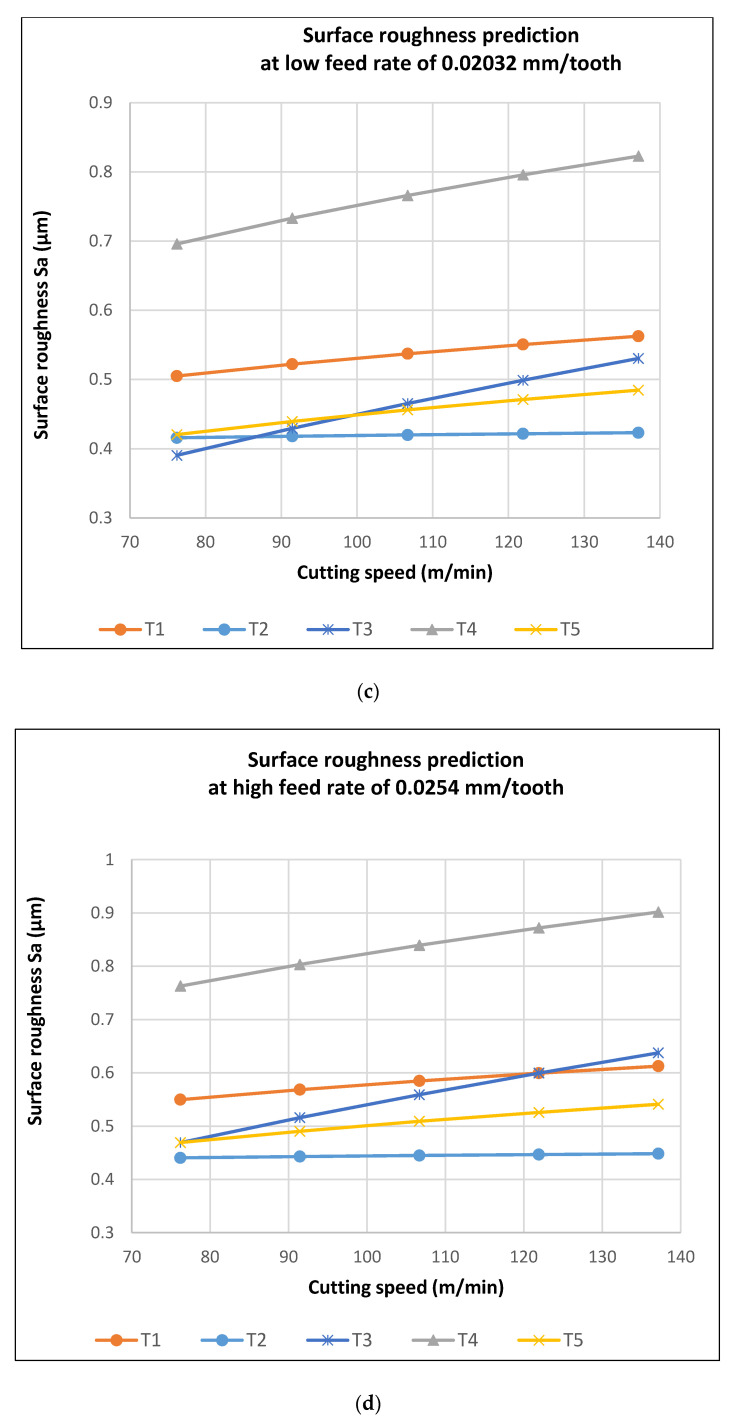
Roughness prediction trends from model equations. (**a**) at low speed of 76.2 m/min (**b**) at high speed of 137.16 m/min (**c**) at low feed of 0.02032 mm/tooth (**d**) at low feed of 0.0254 mm/tooth.

The power form equations for both arithmetic and RMS roughness parameters for the five tools are shown in Table 7. From the equations, the roughness prediction trends in Figure 11 were estimated.

The prediction trends from the regression equations indicated a higher roughness value for Tool 4 with a diameter of 4.75 mm. Increased roughness was also seen at a low tool diameter of 1.194 mm for Tool 1. The charts also indicated the increased sensitivity of Tool 3 with a diameter of 3.175 mm to both cutting speed and feed rate. This is depicted with a steeper trend in prediction. From the statistical analysis, the lowest attainable roughness could be obtained with Tool 3, which had a diameter of 3.175 mm at the lowest cutting speed and feed rate.

### 4.2. Experimental Comparison of Hypereutectic RS Al–50%Si with 6061-T6

**Figure 12:**
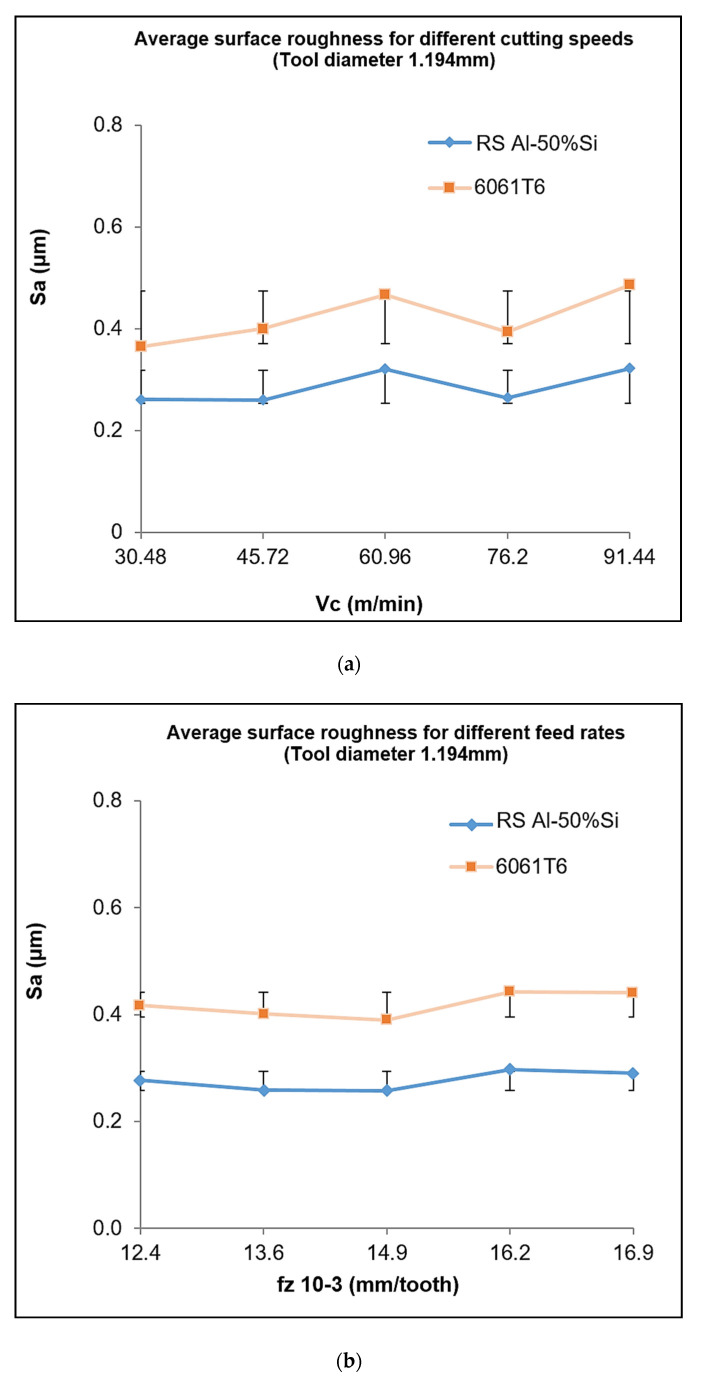

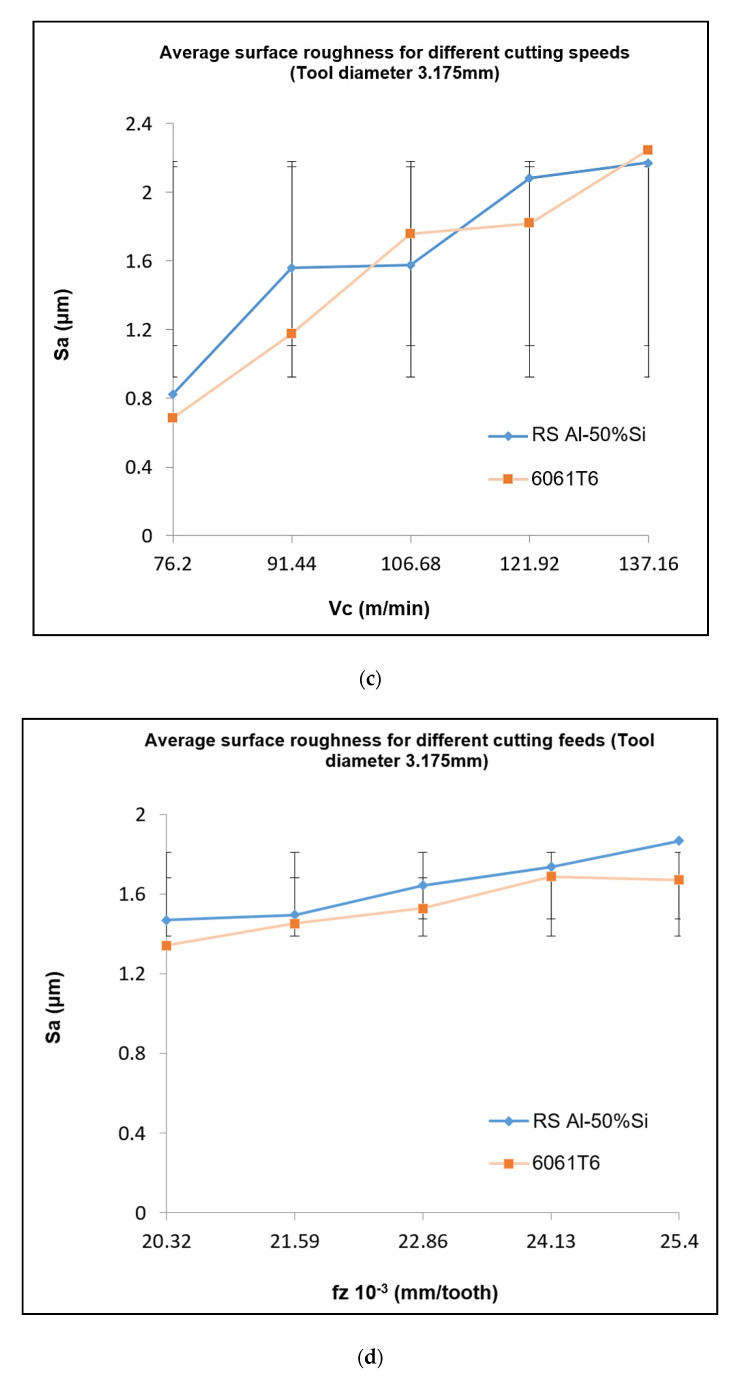
Average arithmetic roughness plots (**a**) at varying speeds for tool T1, (**b**) at varying feeds for tool T1, (**c**) at varying speeds for tool T3, (**d**) at varying feeds for tool T3.

Figure 12a–d shows the average roughness analysis of one parameter while varying the other cutting parameter. A comparison with conventional 6061-T6 aluminum was also performed. 

Figure 12a,b shows the results for Tool 1 (diameter = 1.194 mm). The average roughness curve for the different cutting speeds indicates a much-improved roughness value for the RS Al–50%Si alloy. The average roughness of the cutting speeds for the varying feed rates (Figure 12b), showed improvement in roughness using the hypereutectic alloy. Similar conditions were observed with the other tools, but the results of Tool 3 moderately varied from this trend (Figure 12c,d). In Figure 12c,d, a marginally higher average roughness for the RS Al–50%Si alloy can be observed. From these figures, it can be seen that the roughness was significantly high at increased cutting speeds but decreased when lower cutting parameter values were used. However, the two curves show that the effect of the cutting speed was greater on the surface integrity than the feed rates. The increase in roughness with the tool is believed to be associated with the poor chip formation during cutting. In this cutting condition, an increase in cutting speeds was associated with the defective formation of cutting chips [6]. For this tool diameter, poor chip breakage led to the return of a coiled chip on the surface and between the tool tip and machined surface, thus yielding a deterioration of the surface and highlighting the need to incorporate the chip breakage technology on selected tools. On the other hand, the significant difference in additive elements, the mechanical characteristics, and microstructure between the RS Al–50%Si and Al6061-T6 alloys indicated a major difference in achieved roughness. However, certain parameters’ settings could promote the deterioration of roughness in Al–Si to reflect the conditions seen with the 6061-T6 alloy.

**Figure 13:**
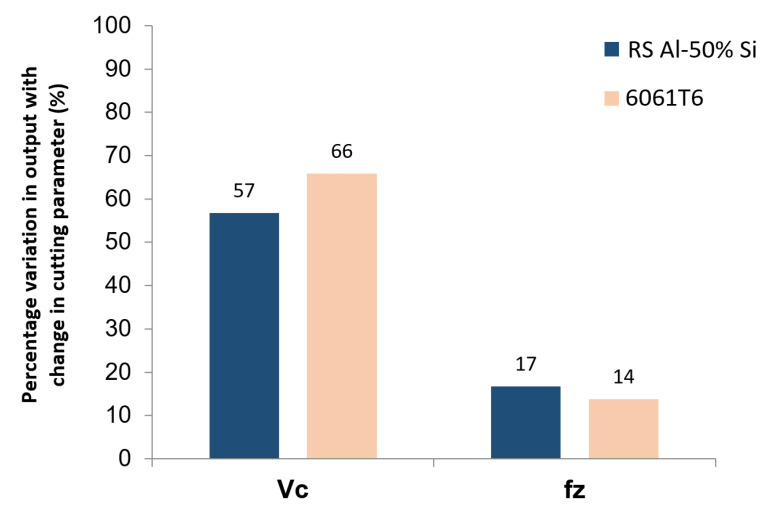
Percentage variation in roughness with a change in the cutting parameter and workpiece materials.

By comparing the percentage variation in response for Tool 3 in Figure 13, a decrease of 57%, for the hypereutectic RS Al–Si alloy with a reduction in cutting speed versus a decrease of about 17% with a reduction in feed rate can be seen. A similar result was also noticeable for Al6061-T6 with an effect of 66% for speed and 14% for feed rate when these two parameters were changed. These differences showed the significant effect of the cutting speed on the surface finish compared to that of the feed rate. Therefore, we can conclude that the cutting speed was the most influential factor, because the more it decreased, the more the roughness decreased (Figure 13). Such results could also have been a consequence of vibrations, indicating the precautions that should be taken to select appropriate and vibration-free speeds (using, e.g., stability lobes).

From the complete comparative analysis between the RS Al–50%Si and Al6061-T6 alloys, of the effect of cutting parameters on the surface roughness, it could be seen that the RS Al–50%Si alloy showed a general improvement in surface roughness measurements. A lower cutting speed and feed rate produced a corresponding reduction in roughness. Greater sensitivity in roughness values to changes in parameters was obtained in the 6061-T6 alloy; this was due to the generally higher roughness from the difference of its microstructure. 

### 4.3. Analysis of Vibration 

**Figure 14:**
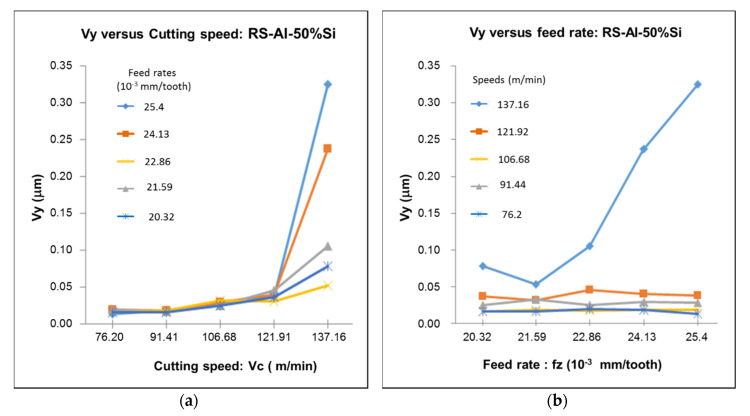
Evolution of the vibration as a function of the variation of (**a**) cutting speed and (**b**) feed rate for the RS Al–50%Si alloy.

**Figure 15:**
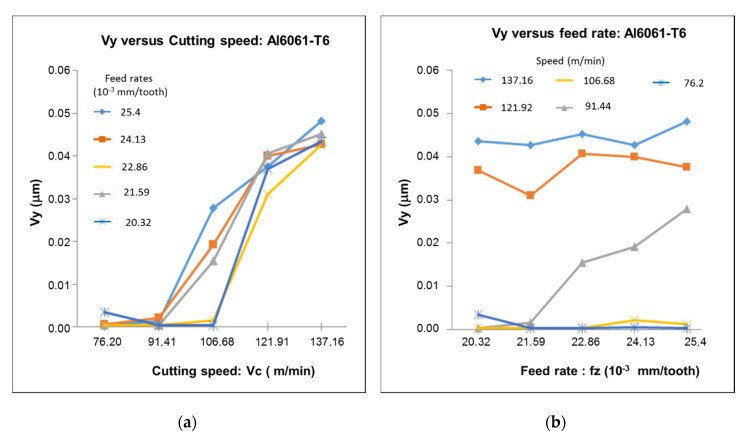
Evolution of the vibration as a function of the variation of (**a**) cutting speed and (**b**) feed rate for the Al6061-T6 alloy.

A significant difference was observed from the influence of the cutting speed and feed rate on the vibration (see Figure 14 and Figure 15). The feed rate appeared to have a smaller contribution to the amplitude of the vibration peaks than the cutting speed. The vibration trend was explained by the existence of thermal and mechanical phenomena occurring in the cutting zone, as well as the characteristics of the material. Based on the hardness of the material and the excessive rubbing action generated at high cutting speeds, high vibrations were expected to be generated during the cutting operation. This was due to the combined effect of plastic deformation and poor chip formation from the high temperature at the cutting zone.

Following the analysis of the vibration (Figure 14 and Figure 15), it was found that the cutting speed considerably influenced the vibration and consequentially further affected the achievable surface finish. An increase in cutting speed caused the tool to vibrate and reduce surface quality. However, this variation in parameter did not negatively affect the final microstructure of the workpiece. The properties of the material were also found to influence vibrational results. The increase in hardness observed in the 6061-T6 samples indicated that the material experienced greater vibrations at lower cutting speeds than the RS Al–50%Si alloy. Below a speed of about 130 m/min, the RS Al–50%Si alloy presented a stable machining condition with limited vibrations. Furthermore, an increase in feed rate produced a negligible influence on vibrational results at these speeds. However, at speeds above 130 m/min, a corresponding increase in feed rate yielded an increase in vibrations. It can be seen that vibration had a more significant importance in the machining of RS Al–50%Si than that of the Al6061-T6 alloy, with clear influences of cutting parameters. It was also observed that a whole correlation could be made between cutting parameters and surface roughness with the RS Al–50%Si alloy; this was not the case with the 6061-T6 alloy. 

### 4.4. Evaluation of Microstructure Change after Machining

**Figure 16:**
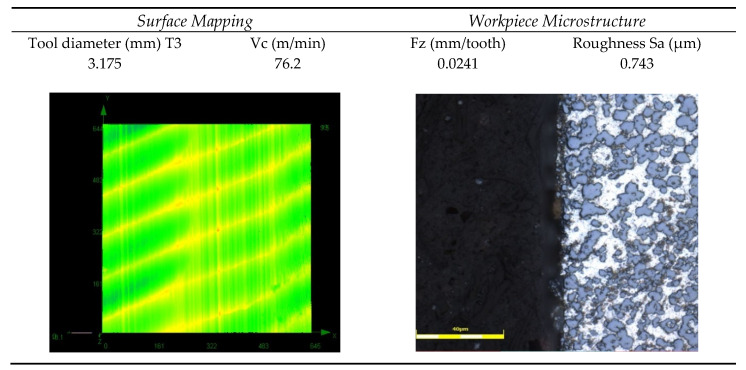
Surface map and microstructure (Tool 3).

After machining tests (with different tools and cutting parameters), observations on the microstructure were made to determine their effect on the contact surface of the tool material [22]. Figure 16 shows the obtained surface map and microstructure when using Tool 3. Two types of microstructural defects were examined: the delamination (cracks between the silicon grains and the aluminum matrix) and the irregularities or the tearing off of silicon grains from the material of the part. 

**Figure 17:**
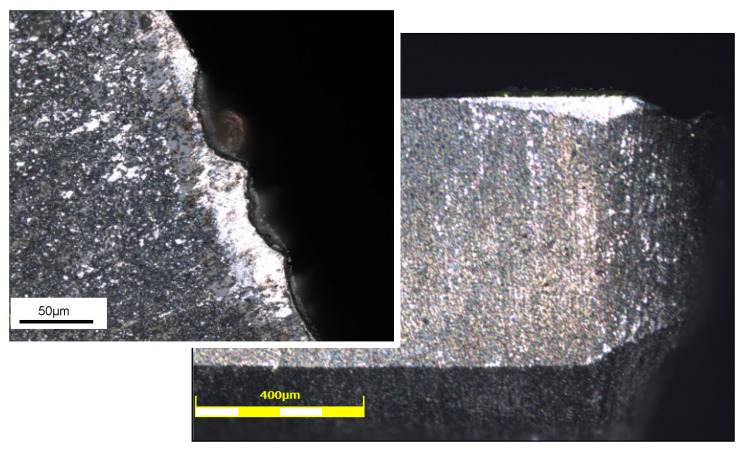
Wear observed on the cutting tool [22].

An overview of the microstructural examination of the alloy after machining revealed no cracks between the silicon grains and the aluminum matrix, as well as no irregularities or the tearing off of silicon grains from the alloy (Figure 16). Machinability challenges were mostly associated with poor tool life. This came as a result of the hardness of the silicon present in the matrix, which also increased mechanical properties such as the strength and hardness of the material, thus promoting rapid tool wear [9]. The wear mechanisms observed on cutting tools were mostly flank wear and microchipping (Figure 17). However, the prominence of silicon in the alloy gave it an added fluidity and a low shrinkage, consequently producing a good material with good castability and weldability.

For samples machined with the recommended cutting parameters (Table 4), the microstructure in the cutting area was studied. These microscopic observations were used to observe the condition of the alloy after machining and to avoid defects that could cause problems in the operation of the final part. During the study, three validation samples were machined at our industrial partner, and the results showed that the maximum errors did not exceed 14.25% [1,2].

## 5. Conclusions

In this study, experimental milling tests were performed on a rapidly solidified hypereutectic Al–Si alloy with a prominent silicon content (over 50%) to identify inherent surface quality challenges faced during industrial machining of these alloys. The effects of cutting parameters such as cutting speed and feed rate on surface roughness were analyzed. This study led us to the following conclusions:

➢ The increase in cutting speed and the feed rate was correlated with a corresponding increase in surface roughness in this material.
➢ Improved machining conditions were observed at cutting speeds below 120 m/min and feed rates below 0.0022 mm/tooth during the machining of the RS Al–50%Si alloy. At cutting speeds below 120 m/min, a stable machining state was achieved with the hypereutectic alloy; however, at cutting speeds beyond this point, a high level of vibration was encountered in its machining. The cutting feed rate was relatively insignificant in vibration responses at low speeds, but further increases in feed rates at speeds above 130 m/min increased cutting vibrations.
➢ The influence of the cutting speed on the roughness was about 50% greater than that of the feed rate.
➢ A comparative analysis of the surface with the conventional Al6061-T6 alloy showed an improvement in surface roughness measurements using the RS Al–50%Si alloy. Greater sensitivity in roughness values to changes in parameters was obtained in the 6061-T6 alloy; this was attributed to its coarser microstructure.
➢ An examination of the microstructure of the alloy after machining revealed no cracks between the silicon grains and the aluminum matrix, as well as no irregularities or the tearing off of silicon grains from the alloy. Machining challenges were mostly associated with poor tool life due to the presence of silicon in the aluminum matrix, which promoted rapid tool wear.

## Figures and Tables

**Table 1:** Mechanical properties of the hypereutectic RS Al–50%Si alloys.

Analysis	$R_{e0,2}\;\left(MPa\right)$	$R_{M}\;\left(MPa\right)$	E (MPa)	A%
**Experimental**	150	230	117	0.94
**Theoretical**	165	252	102	1.7

**R_e0,2_**—0.2% offset Yield strength, **R_M_**—Ultimate strength, **E**—Yield strength, ***A***—Percentage Elongation.

**Table 2:** Mechanical properties of other comparable hypereutectic (Al–Si) alloys.

Mechanical Properties	RSP RSA-463	Al–SiC MMC	AlSiC-9
Density (g/cm^3^)	2.47	3.03	3.01
Hardness (BHN)	230	228	190
Yield strength (Mpa)	130	400	430
Modulus of elasticity (Gpa)	117	223	192
Elongation (% strain)	<1	-	0.295
Tensile strength (Mpa)	200	207	550
Thermal conductivity (W/m-K)	125	175	160

**RSA**—Rapidly Solidified Aluminum, **MMC**—Metal Material Composite.

**Table 3:** Experimental plan for five different tool diameters.

Cutting Tools	T1	T2	T3	T4	T5
Diameter of coupe (mm)	1.194	2.286	3.175	4.750	6.350
Radial depth of cut (mm)	0.119	0.229	0.318	0.475	0.635
Axial depth of cut (mm)	3.581	6.858	9.525	12.700	12.700
Max Cutting speed (m/min)	91.74	137.16	137.16	137.16	137.16
	76.20	121.92	121.92	121.92	121.92
	60.96	106.68	106.68	106.68	106.68
	45.72	91.44	91.44	91.44	91.44
Min Cutting speed (m/min)	30.48	76.2	76.2	76.2	76.2
Max Feed rate (mm/tooth)	0.0169	0.0188	0.0251	0.0376	0.0503
	0.0162	0.0178	0.0241	0.0368	0.0495
	0.0149	0.0165	0.0229	0.0356	0.0483
	0.0136	0.0152	0.0216	0.0343	0.0470
Min Feed rate (mm/tooth)	0.0124	0.0140	0.0203	0.0330	0.0457

Manufacturers’ recommended tool parameters for hypereutectic materials with 12 < % Si < 16.

**Table 4:** Optimal parameters for surface roughness for a max value for 0.8 µm.

	Parameters	For Max 0.8 Roughness		Minimal Registered Roughness	
		RS Al–50%Si	Al6061-T6	RS Al–50%Si	Al6061-T6
**Factors**	Speed Vc (m/min)	76.2	91.44	76.2	91.44
	Feed rate Fz (10^−3^ mm/tooth)	24.13	20.32	22.86	22.86

**Table 5:** Analysis of variance for transformed response for *Sa* (Tool 3).

Source	DF	Seq-SS	Adj MS	F-Value	*p*-Value
**Regression**	2	12.5313	6.26567	241.92	0
**Speed**	1	11.803	0.44038	17	0
**Feed**	1	0.7284	0.72838	28.12	0
**Error**	23	0.5957	0.0259		
**Total**	25	13.127			

**Table 6:** Analysis of variance for transformed response for *Sq* (Tool 3).

Source	DF	Seq-SS	Adj-MS	F-Value	*p*-Value
**Regression**	2	6.101	3.05049	121.69	0
**Speed**	1	5.462	0.44879	17.9	0
**Feed**	1	0.639	0.63902	25.49	0
**Error**	23	0.5765	0.02507		
**Total**	25	6.6775			

**Table 7:** Model equations for surface roughness parameters (*Sa* and *Sq*) for the tools.

Cutting Tools	Surface Roughness Parameters	
	Sa (µm)	Sq (µm)
Tool 1	$Sa=V^{0.184}\times f^{0.38}$	$Sq=V^{0.216}\times f^{0.34}$
Tool 2	$Sa=V^{0.030}\times f^{0.26\;}$	$Sq=V^{0.023}\times f^{0.18}$
Tool 3	$Sa=V^{0.52\;}\times f^{0.80\;}$	$Sq=V^{0.527}\times f^{0.77\;}$
Tool 4	$Sa=V^{0.285}\times f^{0.41\;}$	$Sq=V^{0.321}\times f^{\;0.39}$
Tool 5	$Sa=V^{0.242}\times f^{\;0.49}$	$Sq=V^{0.257\;}\times f^{0.44}$
